# Senate Bill No. 1552

**Published:** 1896-12

**Authors:** 


### SENATE BILL NO. 1552.

Veterinarians must not forget that this bill is still pending before Congress, and there must be no relaxing our efforts if it is to be defeated. Every Senator must be personally appealed to, and he must, if possible, be made to realize that this measure is intended as an entering wedge to retard scientific research and to halt investigations that have already proved a priceless boon to humanity, and if it shall become a law will erect a barrier that will check the agricultural industries in our land, in whose interests so much has been revealed relative to preventive measures for the better control of contagious scourges which for many years have been so disastrous to our live-stock raisers. Much remains to be done in perfecting this and other equally important work, and the voters of every agricultural district should raise their voice against this proposed legislation in these times of depression. Our Government owes a just care and helping hand for the relief of every burden that scientific investigation may lighten and that may enhance the welfare, prosperity, and health of our people. Strike down Senate Bill 1552!

